# Clinical Implications of Fractional Flow Reserve Measured Immediately After Percutaneous Coronary Intervention

**DOI:** 10.1007/s10557-023-07437-0

**Published:** 2023-02-23

**Authors:** Bettina Csanádi, Tamás Ferenci, Gábor Fülöp, Zsolt Piróth

**Affiliations:** 1 Gottsegen National Cardiovascular Center, 29 Haller Str., 1096 Budapest, Hungary; 2 Physiological Controls Group, John von Neumann Faculty of Informatics, Óbuda University, Budapest, Hungary

**Keywords:** PCI, FFR, Outcome, DES

## Abstract

**Purpose:**

The purpose of the present study was to find the independent predictors of Fractional Flow Reserve (FFR) measured immediately after percutaneous coronary intervention with drug-eluting stent implantation (post-PCI FFR) and investigate if applying vessel-specific post-PCI FFR cut-off values to predict target vessel failure (TVF), a composite of cardiac death (CD), non-fatal myocardial infarction (MI) and target vessel revascularization (TVR), or a composite of CD and MI ameliorated its predictive power.

**Methods:**

Consecutive patients with post-PCI FFR measurement at our center between 2009 and 2021 were included in this analysis.

**Results:**

A total of 434 patients with 500 vessels were included. Median pre-PCI FFR was 0.72 with no difference between LAD and non-LAD vessels. Median post-PCI FFR was 0.87. LAD location, male gender, smaller stent diameter, and lower pre-PCI FFR proved to be significant predictors of a lower post-PCI FFR. On a vessel-level, post-PCI FFR, stent length, and diabetes mellitus proved to be significant predictors of TVF and the composite of CD and MI. The best post-PCI FFR cut-off to predict TVF or a composite of CD and MI was 0.83 in the LAD and 0.91 in non-LAD vessels.

**Conclusion:**

LAD location is a predictor of a lower post-PCI FFR. Post-PCI FFR is an independent predictor of TVF as well as of the composite of CD and MI. No uniform target post-PCI FFR value exists; different cut-off values may have to be applied in LAD as opposed to non-LAD vessels.

**Supplementary Information:**

The online version contains supplementary material available at 10.1007/s10557-023-07437-0.

Fractional Flow Reserve (FFR) has become the gold standard in revascularization decisions based on extensive validation, randomized clinical studies, and registries [1–11]. FFR measures the relative epicardial resistance to flow and can be applied not only before percutaneous coronary intervention (PCI), but also after it. Following PCI with an angiographically satisfactory result, one expects the resolution of resistance to flow and cessation of symptoms. Recent studies have however shown that in a significant proportion of cases, residual gradients remain even after satisfactory angiographic result [12–15] leading to low post-PCI FFR. Numerous analyses have provided evidence that FFR measured immediately after PCI carries prognostic implications and lower post-PCI FFR values are linked to poor clinical outcome [16–18]. In most studies, it was also noted that post-PCI FFR values in LAD are significantly lower compared to those measured in non-LAD vessels [15, 17–21], but not in a small study of 66 patients [22]. Post-PCI FFR was shown to be moderately related to major adverse cardiac events (MACE); however, the best cut-off to predict untoward events has been a matter of debate. This may be related to the difference in the studied populations, the pattern of disease (focal or diffuse), but potentially also to the fact that no distinction was made to the different vessels. On the other hand, it has not been firmly established which parameters influenced post-PCI FFR. Hence, we investigated the independent predictors of post-PCI FFR and sought to determine if applying a vessel-specific cut-off of post-PCI FFR ameliorated its predictive power.

## Methods

### Patients

We included all patients who underwent FFR-guided PCI exclusively with drug-eluting stent (DES) implantation and post-PCI FFR measurement at our tertiary care center with at least 1-year follow-up. We excluded only patients who had undergone heart transplantation before PCI and patients in whom the PCI was performed in bypass grafts. Patients could have chronic coronary syndrome (CCS) or acute coronary syndrome (ACS), heart failure, or arrhythmias as an indication of coronary angiography.

The technique of PCI was left entirely to the discretion of the treating physician. FFR was measured using commercially available pressure wires (St. Jude Medical, St. Paul, Minnesota, now Abbott). Hyperemia was achieved mostly using intracoronary boluses of adenosine (200 μg in the left and 100 μg in the right coronary artery) or intravenous infusion of adenosine at the standard dose of 140 μg/kg/min. All patients received guideline-directed medical therapy following PCI.

Of note, all the post-PCI FFR values analyzed were the final ones, after which no further intervention was performed.

The data that support the findings of this study are available from the corresponding author upon reasonable request. The subjects gave informed consent. This study was performed in line with the principles of the Declaration of Helsinki.

### Endpoints

The primary endpoint of the study was target vessel failure (TVF) defined as a composite of cardiac death (CD), non-fatal myocardial infarction (MI), and target vessel revascularization (TVR). The secondary endpoint of the study was the composite of CD and MI. All deaths were considered cardiac unless a clear non-cardiac cause was present. Periprocedural myocardial infarctions related to the index procedure were not counted. Both urgent and elective TVR’s were considered endpoints. Of note, no recoronary angiography was planned. In case a patient had more than one vessel included, the CD was counted as related to all vessels; if a non-fatal myocardial infarction could not be clearly related to a culprit artery, all vessels of the patient in the registry were considered infarct related. Follow-up information was collected from the institutional database, telephone inquiries, and from the database of other centers. In case no follow-up information was available from the above sources, the patient’s unique insurance number was checked in the national death records. Endpoints were analyzed both on a vessel and on a patient level, for the latter, each patient’s lowest post-PCI FFR value was used in case they had more than one vessel with post-PCI FFR measurement included.

### Statistical Analysis

For the multivariable modelling, linear regression was used in the case of post-PCI FFR and the Cox proportional hazards model was used for survival data. In each case, continuous predictors were expanded with splines first, to check if there is any significant nonlinearity [23]. Given the lack of it, linear models were used subsequently. In all cases, multiple imputation with predictive mean matching [24] was used to impute missing data. The Hubert-White method was used for robust covariance matrix estimation in all regression; in the case of vessel-level analysis, clusters were set to patients to account for the correlation of measurements coming from the same patient. Final models are given by presenting beta coefficients (linear regression) or hazard ratios as exponentiated coefficients (Cox regression). Results are shown with 95% confidence intervals. Variables were considered significant if they had *p* ≤ 0.05. Calculations were carried out using the R statistical software package, version 4.2.2.

## Results

**Fig. 1:**
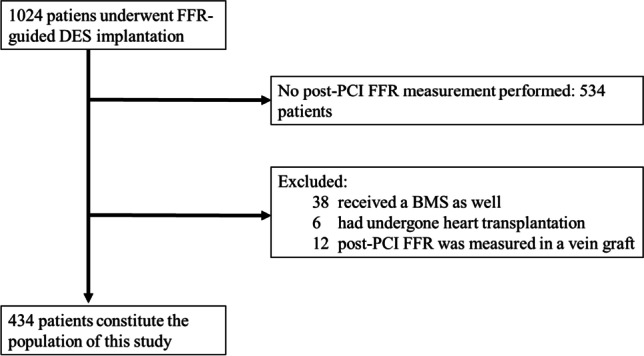
Flowchart of patients

Between March 17, 2009, and January 19, 2021, 1024 patients had FFR-guided DES implantation at our center. Of those, 534 did not have all treated vessels assessed by post-PCI FFR measurement. After excluding those after heart transplantation and graft PCI, 434 patients and 500 arteries were included in our analysis. The flowchart of patients is shown in Fig. 1.

The median age of the patient population was 65 years (IQR: 57–71), 69% were male, 49% had diabetes mellitus; of those, 27% were treated with insulin. Forty-four percent of the patient population had previous PCI and 2% had coronary artery bypass surgery. The characteristics of the patients are summarized in Table 1.

**Table 1:** Characteristics of the patients

Total no. of patients	434
Age—yr (IQR)	65 (57–71)
Male sex—no. (%)	300 (69%)
Hypertension—no. (%)	369 (85%)
Hypercholesterolemia—(%)	321 (74%)
Diabetes mellitus—no. (%)—any	211 (49%)
Treated with insulin	56 (27%)
St/p PCI—no. (%)	192 (44%)
St/p CABG—no. (%)	9 (2%)
LVEF—% ± SD	55±14
LVEF < 50%	117 (27%)

Of the 434 patients, 46 had 2 and 10 had 3 vessels included in our analysis. Of the 500 vessels, 333 (67%) were left anterior descending (LAD), 67 (13%) left circumflex (LCx), and 100 (20%) right coronary arteries (RCA). In 77 cases, the indication of PCI was ACS, in 62, the treated lesion was in-stent restenosis.

The distribution of pre-PCI FFR values is shown in Supplemental Fig. 1. Median pre-PCI FFR was 0.72 (IQR: 0.65–0.77), and no difference was found between LAD and non-LAD vessels (0.72 (IQR: 0.66–0.76) and 0.72 (IQR: 0.63–0.77]), *p* = 0.3011, respectively).

The number of implanted stents was 1.40 per vessel: 1 in 326 vessels (65.2%), 2 in 147 vessels (29.4%), and 3 in 27 vessels (5.4%).

**Fig. 2:**
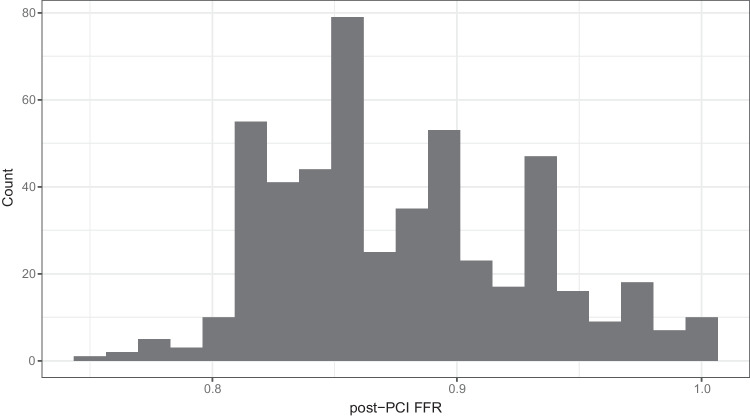
The distribution of post-PCI FFR values

The distribution of post-PCI FFR values is shown in Fig. 2. Median post-PCI FFR was 0.87 (IQR: 0.84–0.91). A post-PCI FFR of ≤ 0.80 was found in 21 arteries (4%), whereas a post-PCI FFR of > 0.95 was found in 44 arteries (9%). LAD vessels had lower post-PCI FFR value than non-LAD vessels (0.85 (IQR: 0.83–0.89) vs. 0.92 (IQR: 0.88–0.94), *p* < 0.001).

The distribution of the difference between post and pre-PCI FFR values (ΔFFR) is shown in Supplemental Fig. 2. LAD vessels had lower ΔFFR than non-LAD vessels (0.14 (IQR: 0.10–0.21) vs 0.19 (IQR: 0.15–0.29), *p* < 0.001).

### Predictors of Post-PCI FFR

**Fig. 3:**
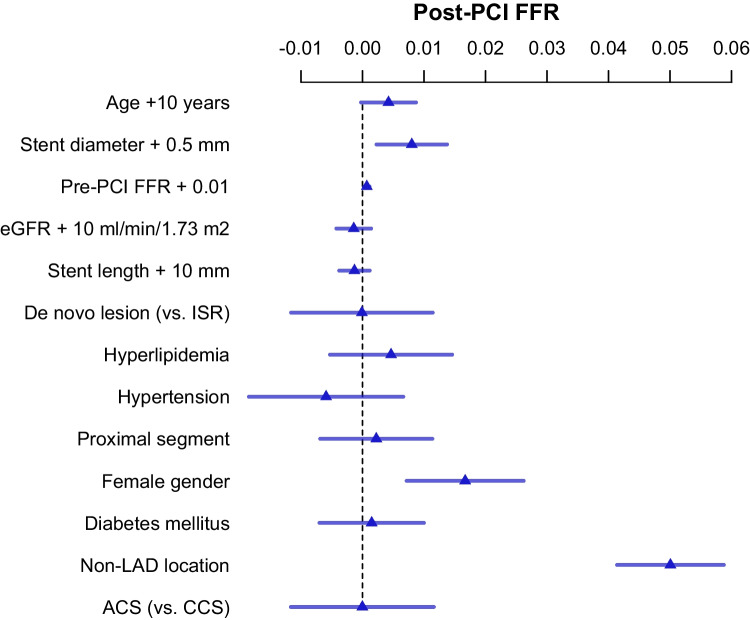
Predictors of post-PCI FFR

By multivariable regression analysis, LAD location (*p* < 0.001), male gender (*p* < 0.001), smaller stent diameter (*p* = 0.006), and lower pre-PCI FFR (*p* = 0.003) proved to be significant predictors of lower post-PCI FFR. Of note, post-PCI FFR measured in non-culprit vessels in ACS and in CCS did not differ significantly, neither had the in-stent restenosis vs. de novo lesion category or diabetes mellitus any significant influence on post-PCI FFR. This is shown in Fig. 3.

### Follow-Up

After a median follow-up of 37 months (IQR: 20–61), survival status is known in 433 of 434 patients (99.8%), whereas follow-up is complete for MI and TVR in 423 (97.5 %). During this period, 27 patients suffered CD, 20 had MI, and 52 TVR occurred. Of the 52 TVR, 10 were performed because of acute myocardial infarction, and 13 were FFR-guided (i.e., the FFR was ≤ 0.80 before TVR was performed). In 4 cases, the lesion was subtotally occlusive which the operator felt to be functionally significant, whereas in 25 cases the revascularization was purely angio-guided. In the latter group, FFR measurement could have resulted in the deferral of TVR. In all, 73 patients suffered TVF (17%), whereas 39 had MI or CD during follow-up (9%).

### Vessel-Level Analysis

**Fig. 4:**
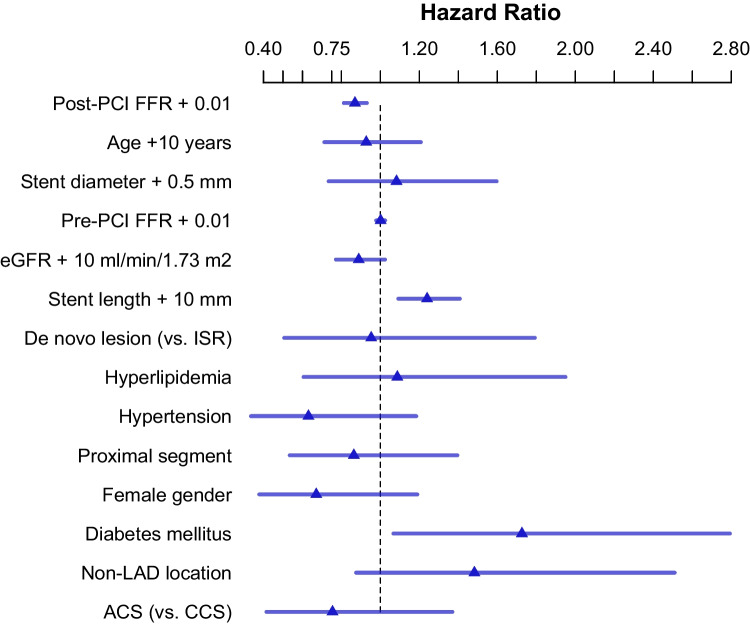
Significant predictors of TVF

**Fig. 5:**
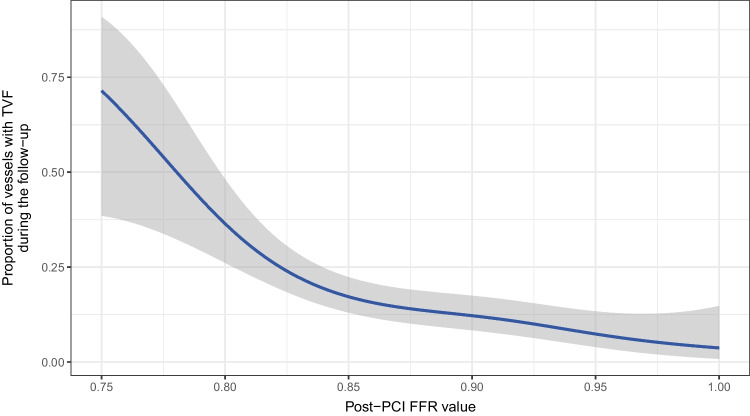
Relationship between TVF and post-PCI FFR

On a vessel-level, post-PCI FFR (*p* < 0.001), stent length (*p* < 0.001), and diabetes mellitus (*p* = 0.026) were found to be significant predictors of TVF (Fig. 4). The frequency of TVF in vessels with post-PCI FFR strata of ≤ 0.80, 0.81–0.85, 0.86–0.90, 0.91–0.95, and > 0.95 were 47.6%, 21.4%, 13.3%, 12.6%, and 2.3%, respectively. The relationship between TVF and post-PCI FFR as a continuous variable is shown in Fig. 5.

By univariate ROC analysis, a post-PCI FFR of 0.83 was found to be the best cut-off to predict TVF according to the Youden-index with a sensitivity of 45%, specificity of 86%, and an area under the curve (AUC) of 0.70.

Given that the median post-PCI FFR values in the LAD were 0.07 units lower than in non-LAD vessels, we also studied these two territories separately. Two-thirds of the studied vessels were LAD, in these, the best post-PCI FFR, by univariate ROC analysis, was also 0.83 according to the Youden-index (sensitivity 60%, specificity 82%, AUC 0.75), whereas in the non-LAD vessels, accounting for one-third of the cases, the best post-PCI FFR to predict TVF was 0.91 according to the Youden-index (sensitivity 65%, specificity 61%, AUC 0.59).

The secondary endpoint of our study was the composite of CD and MI. Its independent predictors were post-PCI FFR (*p* < 0.001), stent length (*p* < 0.001), non-LAD location (*p* = 0.0026), and diabetes mellitus (*p* = 0.015), see Supplemental Fig. 3. The relationship between CD and/or MI and post-PCI FFR as a continuous variable is shown in Supplemental Fig. 4.

By univariate ROC analysis, a post-PCI FFR of 0.83 was found to be the best cut-off to predict the composite of CD and MI according to the Youden-index with a sensitivity of 54%, specificity of 86%, and an AUC of 0.75. In LAD vessels, the best cut-off to predict CD and/or MI according to the Youden-index was also 0.83 with a sensitivity of 72%, specificity of 82%, and an AUC of 0.84. In non-LAD vessels, the best cut-off to predict CD and/or MI according to the Youden-index was 0.91, with a sensitivity of 70%, specificity of 61%, AUC 0.60.

### Patient-Level Analysis

On a patient level, post-PCI FFR (*p* < 0.001) and stent length (*p* = 0.00384) were found to be independent predictors of TVF. The frequency of TVF in patients with the single lowest post-PCI FFR strata of ≤ 0.80, 0.81–0.85, 0.86–0.90, 0.91–0.95, and > 0.95 were 47.6%, 22.2%, 11.7%, 12.5%, and 2.9%, respectively. The relationship between TVF and the single lowest post-PCI FFR as a continuous variable is shown in Supplemental Fig. 5. Note that this figure uses the whole follow-up for each patient, i.e., it neglects the inter-patient differences in follow-up time.

By univariate ROC analysis, a single lowest post-PCI FFR of 0.83 was found to be the best cut-off to predict TVF according to the Youden-index with a sensitivity of 51%, specificity of 85% and an AUC of 0.73.

The independent predictors of the composite of CD and MI on a patient level were post-PCI FFR (*p* < 0.001), stent length (*p* < 0.001), and diabetes mellitus (*p* = 0.03377). By univariate ROC analysis, a single lowest post-PCI FFR of 0.83 was found to be the best cut-off according to the Youden-index to predict CD and/or MI with a sensitivity of 62%, specificity of 85% and an AUC of 0.81 on a patient-level.

## Discussion

In the present analysis, we report on our center’s long-term experience of post-PCI FFR. Our salient findings are as follows. Post-PCI FFR is influenced by the location of the lesion (LAD vs non-LAD), gender, stent (vessel) diameter, and to a minor degree, albeit statistically significantly, pre-PCI FFR. Second, on a vessel-level, only post-PCI FFR, stent length, and diabetes mellitus predicted TVF. We also found different best cut-off values in LAD vs non-LAD vessels. Last, importantly, post-PCI FFR, along with diabetes mellitus, and stent length independently predicted “hard” end points (CD and MI).

Numerous studies have reported on the prognostic value of post-PCI FFR to predict vessel-related MACE from the bare metal stent (BMS) [25] as well as the DES era [17, 18, 26]. Some have reported conflicting findings on its predictive value [12, 13, 27–29]. The reason of heterogeneity of the findings of the studies is speculative and includes the length of follow-up, patient population, and lack of statistical power. A study-level meta-regression analysis by Rimac et al. [30] found an inverse relationship between post-PCI FFR and MACE, and a large, patient-level meta-analysis of Johnson et al. [31] showed an inverse relationship between the post-PCI FFR and the rate of untoward events. Of note, both studies included patients with BMS as well as DES implants and BMS are known to lead to higher TVR and therefore, the conclusions of these analyses may be less pertinent to our present-day practice of implanting only DES.

Only few of the previous reports reported on the power of post-PCI FFR to predict CD and MI because the majority had TVF or MACE as the studied end point which included TVR that usually outnumbered CD and MI by far. Some found that a low post-PCI FFR was associated with a higher rate of CD and MI [12, 16], whereas in the DK CRUSH-VII Registry Study the authors found a statistically significant difference in death between those with a post-PCI FFR > 0.88 vs those with lower than that based on 2 vs 6 such events, but the difference in MI was not statistically significant in the same study (0 vs 2 events) [32]. On the contrary, in the randomized controlled FAME and FAME 2 studies, post-PCI FFR was not found to be correlated with either CD or MI, only TVR [18].

Hwang et al. [21] also suggested that different cut-off values may need to be applied for LAD vs. non-LAD vessels to predict TVF, but because of the low number of CD and MI in their study, no conclusion could be drawn about cut-offs to predict “hard” end points. Also, in contrast to our cohort, pre-PCI FFR values in LAD were lower than in non-LAD vessels.

FFR has gained widespread acceptance in the indication of PCI, and it is generally assumed that an “FFR-guided” PCI—usually meaning the measurement of FFR at a distal spot in the artery of interest before PCI and performing the intervention angiography-guided—will result in the elimination of epicardial resistance to flow, myocardial ischemia, and anginal symptoms. An increasing number of reports attest to the fact that this is far from being the reality in a sizable proportion of cases, also reflected by the high percentage of patients returning for recoronary angiography because of persistent symptoms after technically successful PCI. This may be caused by microvascular angina or extracardiac causes as well as by a failure to relieve significant epicardial resistance to flow.

Post-PCI FFR measurement quantifies the remaining epicardial reduction of maximally achievable flow after revascularization which may be related to diffuse disease or focal, angiographically mild-looking lesions of the untreated segments, or stent-related issues (gross underexpansion, potentially malapposition, geographical miss, edge dissections, tissue protrusion, etc.). Technically, drifts can influence the measured FFR value after PCI and a contralateral chronic total occlusion with retrograde filling from the index vessel can influence post-PCI FFR to a variable degree [33]. These different mechanisms could be elucidated by hyperemic pull-back recording at the end of the procedure which is seldom performed. Likewise, pre-PCI FFR is usually applied as a “snapshot,” reflecting the total amount of epicardial resistance proximal to the sensor of the pressure wire, and in most cases, no attempt is made to elucidate the relative contribution of the different segments which could potentially influence the procedural plan. The concept of 2-dimensional FFR has been put forward: hyperemic pullback recording not only shows the severity but also the distribution of plaques [34].

Does it matter whether a post-PCI FFR of 0.83 is related to diffuse or focal disease? Emerging evidence suggests that it does. A higher pressure drop across a plaque may be related to plaque fatigue and rupture leading to ischemic events [35]. However, as stated above, this is usually not clarified, and this may be one of several reasons why the predictive value of post-PCI FFR and its cut off to predict TVF has been different among published reports.

The report by Collet et al. [36] attest to that angiography is inaccurate in assessing the physiological pattern of coronary artery disease (focal or diffuse). Applying the PPG index (Pullback Pressure Gradient), these authors showed that 36% of the cases were reclassified.

All the above-mentioned factors must be considered when the meaning and actual value of post-PCI FFR are contemplated. Our analysis provides one further piece of evidence: the importance of the vessel. We demonstrated that even though pre-PCI FFR values did not differ between LAD and non-LAD vessels, post-PCI FFR is significantly lower in the LAD, and we found that post-PCI FFR cut-off values to predict TVF as well as a composite of CD and MI were different in LAD vs. non-LAD vessels. The explanation behind this is speculative yet, and includes the fact that LAD usually perfuses a larger myocardial mass making the remaining disease functionally more relevant and emerging evidence supports that hydrostatic pressure differences also play a role [37]. The latter is related to the fact that the middle and distal segments of the LAD in a supine patient run higher than the aortic root where the catheter measures the aortic pressure; therefore, the pressure measured by the sensor of the PressureWire is lower [37]. Before PCI, most of the hyperemic gradient stems from the disease and the hydrostatic pressure difference is negligible, whereas, after successful PCI, the gradient caused by significant stenoses is eliminated making the hydrostatic pressure difference more relevant.

In all, post-PCI FFR can be viewed as a safety net at the end of an angiographically successful PCI indicating a suboptimal functional result which has implications not only for TVR but also for hard endpoints like CD and MI independently from other clinical or angiographic variables. Elucidating the cause of a “suboptimal” functional result by hyperemic pullback recording [12] or intravascular imaging [38] may allow for improving not only the post-intervention physiology but also the clinical outcome.

### Study Limitations

Several limitations of the present analysis must be noted. First, it is a single-center cohort of a limited number of patients. Including patients who received only DES reduced the number of subjects and vessels but made our cohort more homogeneous. Some received first generation whereas the majority had second, and third-generation DES implanted, we did not evaluate the effect of this difference. The cause of death could not be firmly established in all cases, these were considered cardiac deaths. TVR was sometimes performed without FFR measurement, only based on angiography. Events were not adjudicated by an independent Clinical Event Committee. We could not study the anginal status of our patients, hence could not establish the relationship between post-PCI FFR and quality of life. PPG was not measured, and no systematic pull-back recording was performed; therefore, we could not establish the role of diffuse vs. focal nature of the remaining disease. However, in all cases, we checked for drifts and in case a drift of > 0.03 units was found, we corrected the measured value or repeated the measurement after re-equalizing. Angiograms were not systematically checked for diffuse disease and no intravascular imaging (IVUS or OCT) was performed to establish the diagnosis of diffuse disease not recognized by angiography. Patients and their treating physicians were aware of the measured post-PCI FFR; however, all interventions were considered angiographically successful and only a small minority of vessels had a post-PCI FFR of ≤ 0.80.

## Conclusions

In conclusion, we found that post-PCI FFR was an independent predictor of TVF as well as of the composite of CD and MI in an unselected patient population undergoing FFR-guided DES implantation. Moreover, LAD location was found to be a predictor of a lower post-PCI FFR. Our data suggest that no uniform target post-PCI FFR value exists; in order to improve the predictive power of post-PCI FFR, different cut-off values may have to be applied in LAD as opposed to non-LAD vessels.

## Supplementary Information

Supplementary materials 1: Supplemental figure 1

Supplementary materials 2: Supplemental figure 2

Supplementary materials 3: Supplemental figure 3

Supplementary materials 4: Supplemental figure 4

Supplementary materials 5: Supplemental figure 5 (PDF 6 kb)

## Data Availability

The data that support the findings of this study are available from the corresponding author upon reasonable request.
