# Supplementary figures and images for: Clinical Implications of Fractional Flow Reserve Measured Immediately After Percutaneous Coronary Intervention
Source: Cardiovasc Drugs Ther. 2023 Feb 23;38(5):917–25. doi: 10.1007/s10557-023-07437-0 (PMC11438715; doi:10.1007/s10557-023-07437-0)

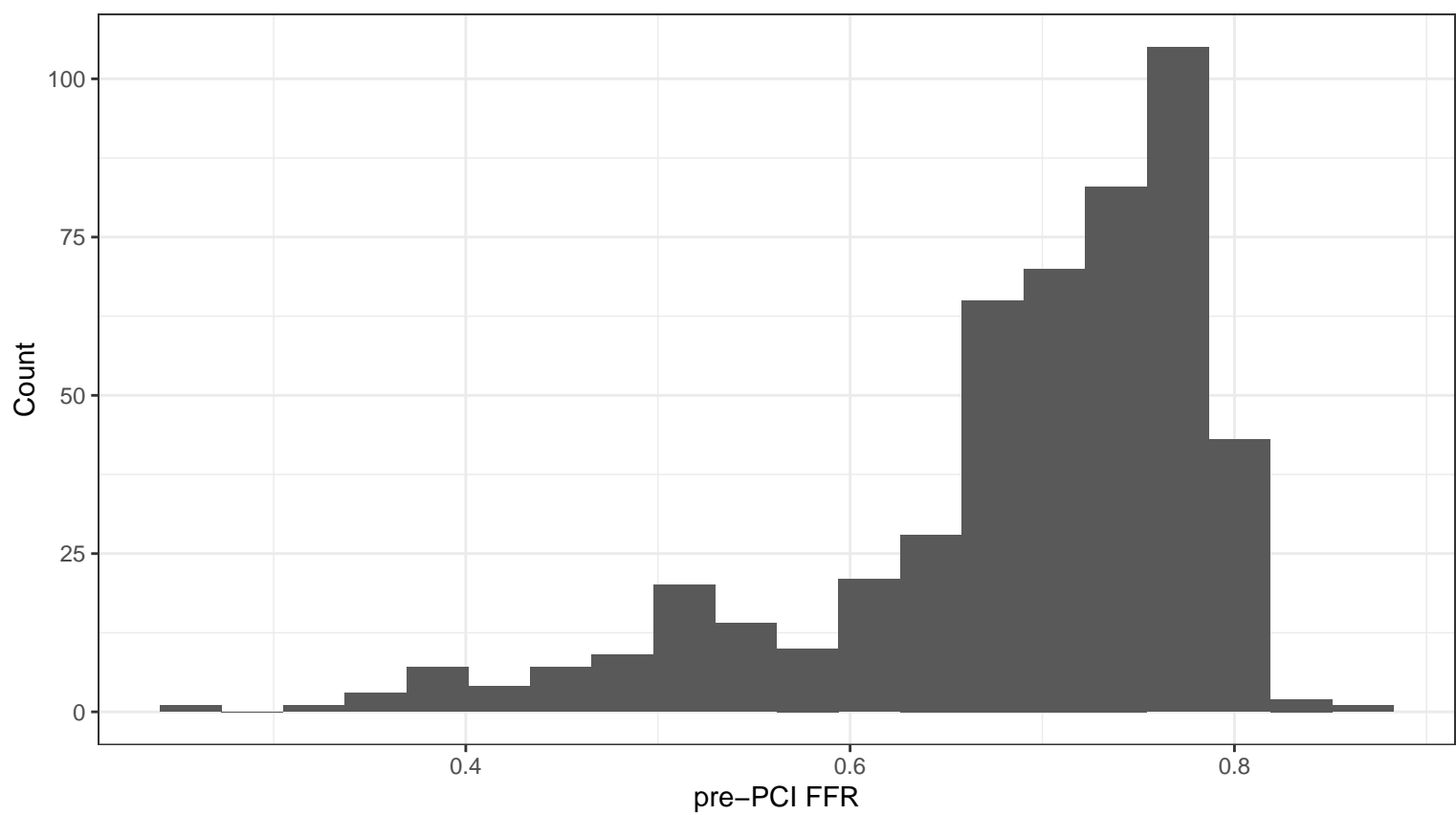

Supplement: Supplementary file 1 — Supplemental figure 1 [file 10557_2023_7437_MOESM1_ESM.pdf]

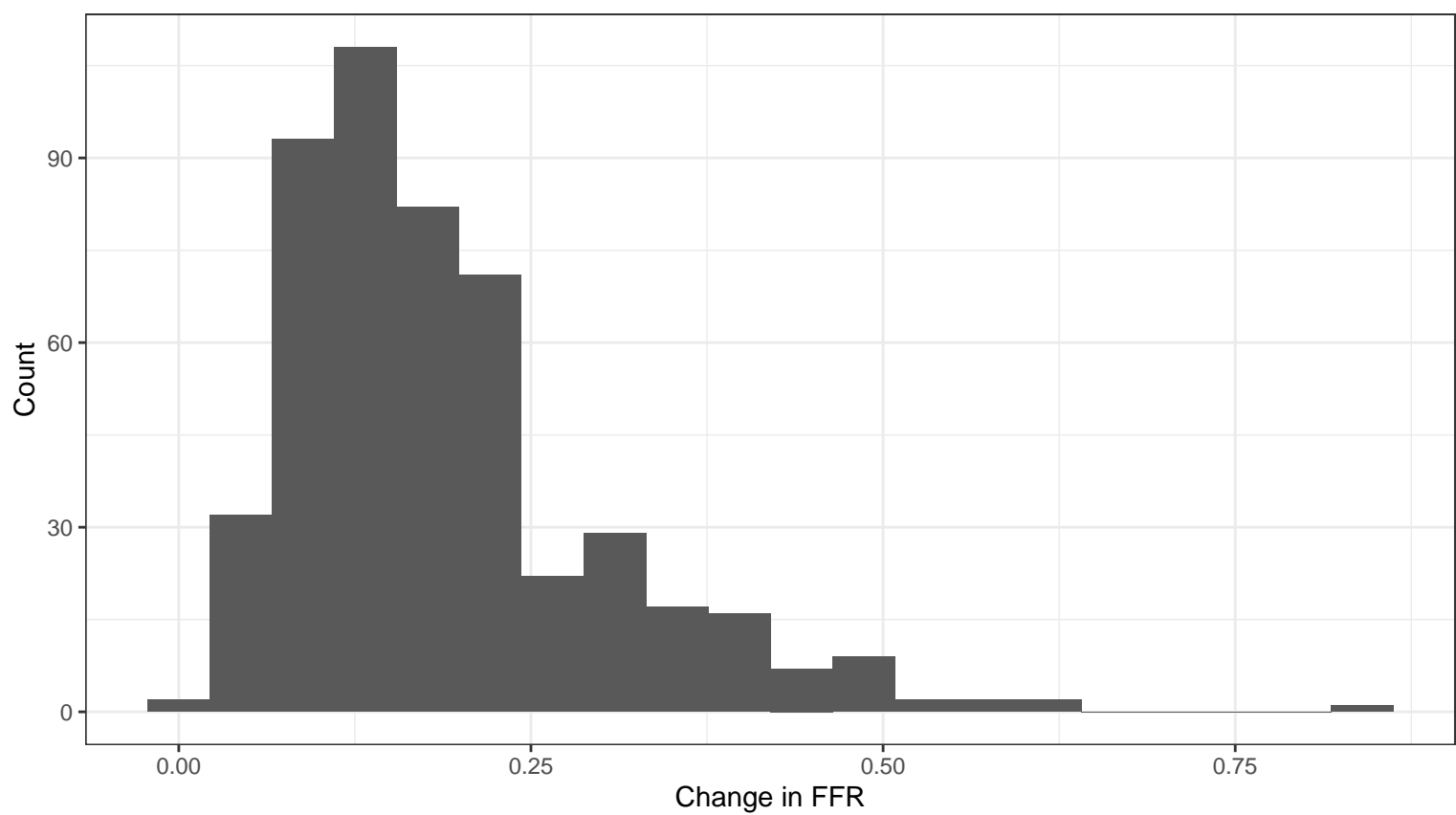

Supplement: Supplementary file 2 — Supplemental figure 2 [file 10557_2023_7437_MOESM2_ESM.pdf]

## Hazard Ratio

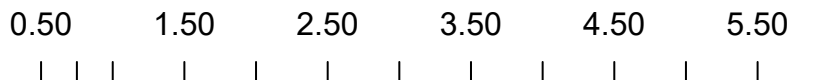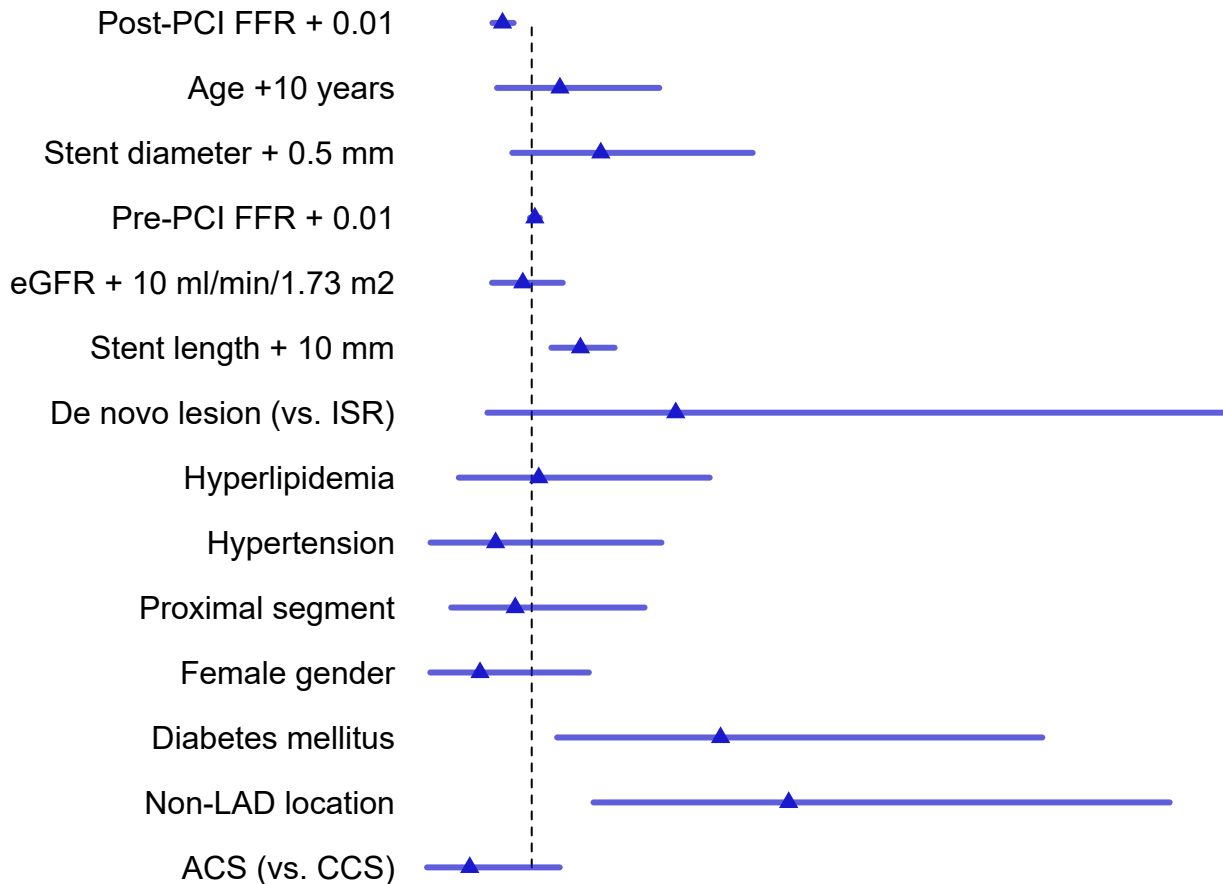

Supplement: Supplementary file 3 — Supplemental figure 3 [file 10557_2023_7437_MOESM3_ESM.pdf]

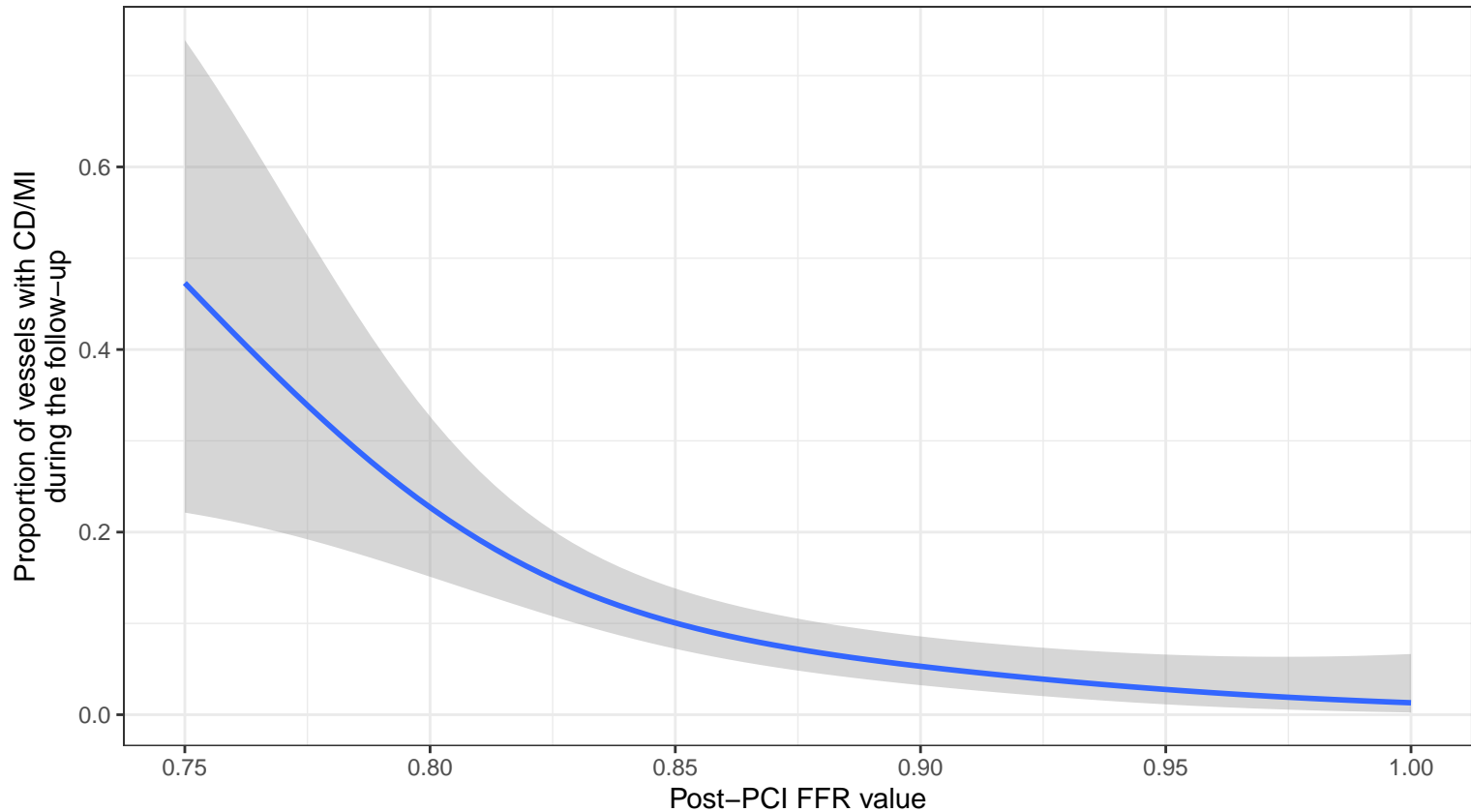

Supplement: Supplementary file 4 — Supplemental figure 4 [file 10557_2023_7437_MOESM4_ESM.pdf]

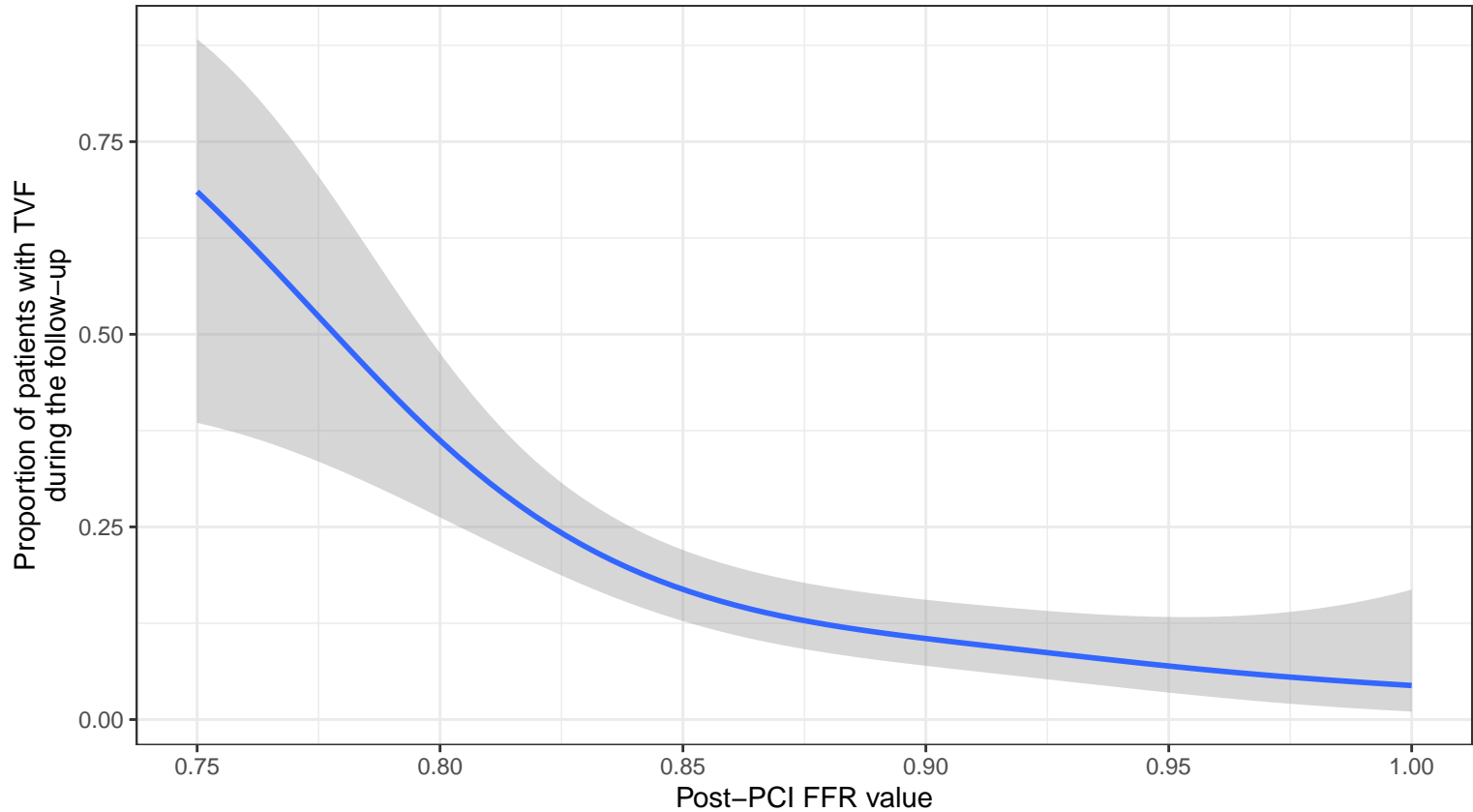

Supplement: Supplementary file 5 — Supplemental figure 5 (PDF 6 kb) [file 10557_2023_7437_MOESM5_ESM.pdf]
